# Small intestinal bacterial overgrowth in irritable bowel syndrome: are there any predictors?

**DOI:** 10.1186/1471-230X-10-23

**Published:** 2010-02-22

**Authors:** Savio C Reddymasu, Sandra Sostarich, Richard W McCallum

**Affiliations:** 1 Department of Medicine, Division of Gastroenterology, Kansas University Medical Center, Kansas City, Kansas, USA

## Abstract

**Background:**

Small intestinal bacterial overgrowth (SIBO) is a condition in which excessive levels of bacteria, mainly the colonic-type species are present in the small intestine. Recent data suggest that SIBO may contribute to the pathophysiology of Irritable bowel syndrome (IBS). The purpose of this study was to identify potential predictors of SIBO in patients with IBS.

**Methods:**

Adults with IBS based on Rome II criteria who had predominance of bloating and flatulence underwent a glucose breath test (GBT) to determine the presence of SIBO. Breath samples were obtained at baseline and at 30, 45, 60, 75 and 90 minutes after ingestion of 50 g of glucose dissolved in 150 mL of water. Results of the glucose breath test, which measures hydrogen and methane levels in the breath, were considered positive for SIBO if 1) the hydrogen or methane peak was >20 ppm when the baseline was <10 ppm, or 2) the hydrogen or methane peak increased by 12 ppm when baseline was ≥10 ppm.

**Results:**

Ninety-eight patients were identified who underwent a GBT (mean age, 49 y; 78% female). Thirty-five patients (36%) had a positive GBT result suggestive of SIBO. A positive GBT result was more likely in patients >55 years of age (odds ratio [OR], 3.6; 95% confidence interval [CI], 1.4-9.0) and in females (OR, 4.0; 95% CI, 1.1-14.5). Hydrogen was detected more frequently in patients with diarrhea-predominant IBS (OR, 8; 95% CI, 1.4-45), and methane was the main gas detected in patients with constipation-predominant IBS (OR, 8; 95% CI, 1.3-44). There was no significant correlation between the presence of SIBO and the predominant bowel pattern or concurrent use of tegaserod, proton pump inhibitors, or opiate analgesics.

**Conclusions:**

Small intestinal bacterial overgrowth was present in a sizeable percentage of patients with IBS with predominance of bloating and flatulence. Older age and female sex were predictors of SIBO in patients with IBS. Identification of possible predictors of SIBO in patients with IBS could aid in the development of successful treatment plans.

## Background

Irritable bowel syndrome (IBS) is the most commonly diagnosed chronic functional gastrointestinal (GI) disorder and has an estimated prevalence of 10% to 15% in North America [1]. Irritable bowel syndrome affects females approximately twice as often as males and is most frequently diagnosed in individuals between the ages of 30 and 50 years [1,2]. Symptoms of IBS can substantially impact patients' quality of life, and the effect of IBS on physical and psychological health can negatively impact the workplace [3-5]. Results of a large US survey (N = 5430) showed that individuals with IBS missed substantially more workdays per year (mean, 13.4 days) compared with individuals without a functional GI disorder (mean, 4.9 days) [6]. Thus, the economic burden of IBS in the United States is substantial and includes estimated direct costs (eg, physician visits) of $1.7 to $10 billion annually and estimated indirect costs (eg, employee absenteeism) of up to $20 billion annually [7].

Irritable bowel syndrome is characterized by abdominal discomfort associated with altered bowel habits including constipation, diarrhea, or alternating periods of constipation and diarrhea. Additional symptoms of IBS may include abdominal bloating and flatulence, but specific GI symptoms often vary among patients. The pathophysiologic mechanisms contributing to IBS are not fully understood, but an emerging hypothesis suggests that small intestinal bacterial overgrowth (SIBO) may contribute to IBS pathophysiology [8]. Small intestinal bacterial overgrowth involves abnormal growth (ie, >10^5 ^colony forming units/mL) of endogenous bacteria in the small intestine resembling those normally found in the colon [9,10]. A potential link between SIBO and IBS has been suggested by symptom similarities (eg, abdominal discomfort, bloating, and flatulence) [8,11,12] and by the reported prevalence of SIBO in patients with IBS [13-17]. In studies using lactulose or glucose breath testing, SIBO was detected in up to 84% (Range 4-84%) of patients who met Rome I [15,17] or Rome II [13,14,16] criteria for IBS. Furthermore, results from multiple clinical studies have shown that treatment with antibiotics can reduce or eradicate SIBO [14,15,17-22] and improve symptoms of IBS [15,17,18,21-24], evidence that further supports the role of SIBO in IBS. Published clinical data evaluating characteristics associated with SIBO in patients with IBS are lacking. Therefore, the purpose of this clinical study was to identify potential predictors of SIBO occurrence in patients with IBS with predominance of bloating and flatulence.

## Methods

Patients ≥ 18 years of age who met Rome II criteria for IBS who had predominance of bloating and flatulence and referred to the Functional Bowel Diseases clinic at a tertiary care University Medical center supervised by one of the investigators (RWM) between 2004 and 2006 were included in the study. The Human Subjects Committee at the Kansas University Medical Center approved the study. Individuals with predisposing conditions for SIBO (eg, diabetes mellitus, scleroderma, prior small intestinal surgery) were excluded. Demographic data, predominant bowel habit pattern, and concurrent use of opiate analgesics, tegaserod, and proton pump inhibitors (PPIs) were recorded at study entry. Patients were subcategorized as having diarrhea-predominant IBS (IBS-D), constipation-predominant IBS (IBS-C), or alternating IBS (IBS-A) based on predominant bowel habits.

Patients who had taken systemic antibiotics or antifungals in the previous month were excluded from breath testing. All other patients with IBS with predominance of bloating and flatulence underwent a glucose breath test (GBT) at study entry. Patients were required to have a low carbohydrate meal the evening before testing and to fast for ≥12 hours before testing. Smoking and physical exercise were not allowed 1 hour before the test was administered. Immediately before the GBT, patients used a mouthwash containing 40 mL of 1% chlorhexidine. Two breath samples were obtained at baseline and at 30, 45, 60, 75, and 90 minutes following ingestion of 50 g of glucose dissolved in 150 mL of water. The baseline and post-glucose ingestion peak values for hydrogen and methane were recorded for each patient, and the total excretion of hydrogen and methane was calculated as an area-under-time concentration curve. Expiratory breath samples were obtained using a commercial device (GaSampler; QuinTron Instrument Company, Milwaukee, Wisconsin) to confirm sampling of alveolar gas. This device allows the separation of the initial 500 mL of dead space air from the remaining alveolar air collected in a gas-tight bag. Samples were analyzed immediately after collection. Gas chromatography (Model DP; QuinTron Instrument Company, Milwaukee, Wisconsin) was employed to measure the concentration in parts per million (ppm) of hydrogen and methane in the air. The breath test result was considered positive for SIBO if: 1) the hydrogen or methane peak was >20 ppm when baseline was <10 ppm or, 2) the hydrogen or methane peak increased by >12 ppm when baseline was ≥10 ppm.

Logistic regression was employed for statistical analysis and odds ratios (OR) with 95% confidence intervals (CI) were calculated for comparisons of a positive breath test result with sex, previous and current use of IBS medication(s), and predominant bowel habits.

## Results

A total of 169 patients (25 male) who met the Rome II criteria for IBS were evaluated in the Functional Bowel Disease clinic during the study period. Ninety eight (76 female) of these patients had bloating and flatulence as the predominant symptoms and underwent testing for SIBO with a GBT. The mean age of patients in this cohort was 49 years (range, 21-85 years; Table 1). Fifty-one patients (52%) had IBS-C, 38 patients (39%) had IBS-D, and 9 patients (9%) had IBS-A.

**Table 1:** Demographic and clinical characteristics of patients with IBS and SIBO

Characteristics	All patients (N = 98)	IBS with SIBO (n = 35)	Odds ratio* (95% CI)
Age >55 y, n (%)	28 (29)	16 (46)	3.6 (1.4-9.0)
Female, n (%)	76 (78)	32 (91)	4.0 (1.1-14.5)
IBS subtype, n (%)			
IBS-C	51 (52)	19 (54)	1.2 (0.5-2.6)
IBS-D	38 (39)	15 (43)	1.3 (0.6-3.0)
IBS-A	9 (9)	1 (3)	0.2 (0.02-1.7)
Medications,** n (%)			
Tegaserod	15 (15)	6 (17)	1.1 (0.4-3.3)
Opiate analgesic	38 (39)	13 (37)	1.0 (0.4-2.3)
Proton pump inhibitor	21 (21)	7 (20)	0.8 (0.3-2.1)

IBS: irritable bowel syndrome; SIBO: small intestinal bacterial overgrowth; CI: confidence interval; IBS-C: constipation-predominant IBS; IBS-D: diarrhea-predominant IBS; IBS-A: alternating IBS.

*Comparing patients with IBS versus patients with IBS and SIBO, **Previous and current use of specific IBS therapy.

A subgroup of 35 patients (36%) was determined to have SIBO based on a positive GBT result, of which the majority of patients were female (n = 32; 91%). Glucose breath testing was more likely to be positive in females with IBS (OR, 4.0; 95% CI, 1.1-14.5; Table 1) and patients >55 years of age (OR, 3.6; 95% CI, 1.4-9.0; Table 1). Hydrogen was the predominant gas detected in patients with IBS-D (OR, 8; 95% CI, 1.4-45), and methane was detected more frequently in patients with IBS-C (OR, 8; 95% CI, 1.3-44). No significant correlation was observed between the presence of SIBO in patients with IBS and predominant bowel pattern and use of opiate analgesics, tegaserod, or PPIs (Table 1). All patients with a positive GBT were treated with antibiotics.

## Discussion

Existing clinical evidence seems to suggest that SIBO may contribute to the pathophysiology of IBS. However, the reported incidence of SIBO varies according to the detection method employed. In studies that included lactulose or glucose breath testing, SIBO was detected in up to 84% of patients who met Rome criteria for IBS [13-17]. Because breath tests indirectly measure bacteria and are associated with relatively low sensitivity and specificity [10], many consider direct bacterial assessment of intestinal aspirate cultures a better method of detecting SIBO [8,9,25]. However, direct sampling also has limitations, including lack of accessibility to the distal small intestine and potential for contamination during sampling [25]. Clinical studies employing direct sampling of jejunal aspirates detected SIBO in 4% to 12% of patients with IBS, a lower prevalence compared with results of breath testing. The most accurate method for detecting SIBO is the subject of much debate [10,12,26]; however, results from clinical studies evaluating SIBO in IBS have confirmed the presence of SIBO in a subset of patients with IBS. Additionally, reports that antibiotic therapy normalized breath test results [14,15,17-22] and improved IBS symptoms [15,17,18,21-24] provide support for the potential role of bacteria in IBS. In our study, a positive GBT was seen in 36% of patients with IBS-like symptoms, especially those with predominance of bloating and flatulence. This group had higher pre-test probability of SIBO, because if the whole cohort of 169 patients are considered and if it is assumed that IBS patients without bloating or flatulence did not have SIBO, then the prevalence of SIBO in IBS in our cohort seems to be lower at around 21%.

The purpose of the present study was to identify specific characteristics that could serve as potential predictors of the association of SIBO development in patients with IBS and not to estimate the prevalence of SIBO in IBS. The incidence of SIBO in our cohort was higher than that reported by other groups who employed the GBT method [26,27]. One explanation could be that the patients who underwent GBT in our study could be regarded as having a higher likelihood of a positive test based on prominence of bloating and flatulence in their symptom profile.

Significant findings of this study are that patients >55 years of age, females with IBS like symptoms, especially those with prominence of bloating and flatulence are more likely to have a positive GBT. Although there is no previously published clinical evidence suggesting SIBO is more common in females, substantially more females than males are diagnosed with IBS accounting for this difference [1,28]. Additionally, SIBO is more prevalent in older individuals as suggested by prior studies [29-31], most likely as a result of reduced intestinal motility with advancing age [32]. Alternatively, no specific bowel pattern was indicative of SIBO in the present study, a surprising observation given that diarrhea has previously been reported as the predominant bowel habit in patients with SIBO [12]. Hydrogen was the predominant gas detected in patients with IBS-D and methane was the gas detected in IBS-C, a finding observed in prior studies.

Finally, no significant correlation was observed between detection of SIBO in patients with IBS and previous or current use therapeutic agents such as opiate analgesics, PPI's, or tegaserod. This lack of correlation was also unexpected given that opiate analgesics are known to slow intestinal motility [33], which could potentiate bacterial overgrowth; tegaserod therapy is associated with substantial acceleration of small intestinal motility and hypothetically, could protect against SIBO [34]; and long-term use of PPIs is theoretically linked to the development of SIBO [35]. A recent article also speculated that since IBS patients are more likely to receive PPI therapy than healthy controls, they are more likely to develop SIBO due to reduction of the gastric acid barrier that prevents bacterial overgrowth in the small bowel [36]. However, we did not find the association between PPI use and a positive GBT in our study. The duration of hypochlorhydria due to PPI's is variable and not sustained and this effect is unpredictable unless PPI's are reliably taken every day by the patient.

It could be pointed out that a sizeable number (38%) of IBS patients in the study were taking opiate analgesics. The reason for this disproportionately high use of narcotic analgesics is because our center is a referral center for functional bowel disease and most of these patients were being prescribed these agents prior to referral to our center.

Some of the limitations of this study include: a) This study being a retrospective review, b) The patients who underwent GBT testing for SIBO were not consecutive patients and included only those with prominence of bloating and flatulence, c) Lack of control arm and absence of data regarding the rate of a positive GBT in healthy controls, and d) Although we found that the GBT was more likely to be positive in patients > 55 years, it is hard to explain whether this is a result of advancing age or related to IBS. Certainly, the patients seen at this tertiary setting had been evaluated by a number of physicians in the past and had the diagnosis of IBS for a number of years, hence SIBO could have been present for some years, including when they were much younger. One logical conclusion would be that GBT is more likely to be positive in a person >55 years of age who has IBS like symptoms, especially those with prominence of bloating and flatulence.

## Conclusion

In summary, a significant percentage (36%) of patients with IBS who have bloating and flatulence as their predominant complaint have a positive GBT suggestive of SIBO. Older age and female sex seem to be predictors for SIBO. The identification of characteristics specific to the subgroup of patients with SIBO and IBS may provide further understanding of the pathophysiologic mechanisms of IBS and contribute to the development of successful treatment strategies.

## Competing interests

The authors declare that they have no competing interests.

## Authors' contributions

SCR conceptualized, helped with data collection and analysis, and drafted the manuscript. SS helped with performing the H2 breath tests and data collection. RWM conceptualized, edited the manuscript for important intellectual content and has read and approved the final version of the manuscript.

## Pre-publication history

The pre-publication history for this paper can be accessed here:

http://www.biomedcentral.com/1471-230X/10/23/prepub
